# History of an Epidemic Typhoid Fever at Somerville, Ohio, Illustrating the Diffusion of the Disease by Contagion

**Published:** 1855-07

**Authors:** Allen A. Barnett

**Affiliations:** Oxford, O.


### Art. II.—HISTORY OF AN EPIDEMIC TYPHOID FEVER AT SOMERVILLE, OHIO, ILLUSTRATING THE DIFFUSION OF THE DISEASE BY CONTAGION.

BY ALLEN A. BARNETT, M. D.

The town of Somerville lies about thirty-five miles North of Cincinnati. The fever of which I propose to give an account, first made its appearance about a mile and a half from the town, in the family of Mr. L. Fox, from which it extended through the neighborhood and to the village. It was confined principally to the farming community, all of them in comfortable circumstances, living in comfortable houses, capable of good ventilation, neat, cleanly, and industrious in their habits.

As to topography, the country is broken and uneven, without marshes, and is watered by a swift-running stream. An unusually healthy year had preceded the outbreak of the epidemic. The winter was mild at its opening; towards the latter part of December the weather was changeable from mild to extreme cold; after the first of January it became a steady, cold season, freezing all the time, attended with unusual dryness for that period of the year.

It was about the first of January that the eldest son of Mr. Fox came home complaining of feeling ill. He had been engaged in teaching school. He remained at home trying, as he said, to work off his sickness. For several days he had pains in his head, back, and limbs; inability to do anything; was inclined to be stupid. He bore up a few days, but at last was compelled to take to his bed, when a physician was called in who pronounced the disease typhoid fever.

In this case the usual symptoms supervened, and the case progressed to about the twenty-third day, when the second son, aged twenty-one years, was taken in the same way. The attack was a mild one, but, through imprudence in eating, he suffered a relapse. These two young men were put in the same room, the family waited upon them, and the neighbors were there occasionally.

The mother of the boys took the fever next, and was sick sixteen days, and died. In a short time four more of the family were taken about the same time, three boys aged seven, fourteen, seventeen, and a daughter aged nine years, and not long after these another son aged nineteen. In two weeks after the latter his brother-in-law and wife, and a niece, who were waiting on the sick ones, took the fever; the brother-in-law, aged forty-seven, after three weeks sickness, died. Three of the children, one of his brother's-in-law, aged seven, and two of his own, one aged seven years, and another aged seven months, were sent away a mile or so to stay, but in two or three weeks all took the fever, the two oldest in a light form and recovered; the babe was sick three weeks and died. A married daughter, aged twenty-seven, living away some distance, came to see the family, was there a part of two days, attended her mother's funeral and went home; four days after she reached home she died.

Another brother-in-law, living a mile and a half from the place, came over one day to see them, was about the house but an hour or so; he took it a short time afterwards. The attack was mild and he recovered, and a friend waiting upon him was also attacked and recovered.

There was not much variation in the symptoms, but all were taken more or less alike, with chills and rigors, the period of access being from two to seven days. Two had bloody discharges, two had involuntary discharges; all had more or less delirium, coma-vigil, and tympanites; several had profuse diarrhœa; all sweated; those that recovered sweated the most. There were four in one room, three in another, two in another, &c. All the rooms were heated with stoves.

The babe, before spoken of, was taken to the town to a brother's; the child was taken sick there, and his daughter, a maiden lady, took care of it; she took it from the child, was sick two weeks and died, aged thirty-five years.

A neighbor and her daughter in the town came in and took care of these two persons; they were both attacked and recovered.

H. Young, a neighbor of Fox No. 1, took the fever in February, also a young man living with him; they were in attendance upon the sick at Fox's. The house was kept well ventilated; both recovered.

Samuel Young, neighbor also to Fox No. 1; his son was in attendance upon the Foxs in February; was very sick, but recovered. A daughter, very robust, weighing over three hundred pounds, took it and died in a few days, aged seventeen; was attended by a Thompsonian. He gave her but *one potion;* another daughter took it and is now convalescent; a son took it from this daughter; the attack was mild; recovered.

E. Davis, neighbor to Fox No. 1, living a mile distant, was waiting upon the sick at Fox's every day for several weeks; was taken sick with the fever on the 13th of February, took to the bed the first day; was not too sick to go about a little for several days; was sick seven or eight weeks. Two weeks after he was taken sick his eldest daughter, aged sixteen, was taken down; was sick six weeks and recovered; his two oldest sons were taken sick soon after the daughter; one sick four, the other five weeks. The first one, through imprudence, had a relapse, and lay five weeks longer; both recovered. Another daughter, aged ten, and a son aged five, were taken about two weeks after the daughter; the attack was mild; both recovered. Two weeks later another daughter, aged twelve, was taken; she was sick six weeks and recovered. The babe, aged ten months, was next taken; attack mild; recovered.

W. Davis, a brother, was with the family waiting upon the sick; went home to Piqua; two weeks after his return home was taken sick; lay four weeks and died; aged twenty-eight.

In this family the period of access was short; the subjects were all troubled with coma-vigil. Two had involuntary discharges, three had muttering delirium, two had loss of speech; there were eight attacked in the family; all recovered.

A boy named Gingery, aged fourteen, living on the farm of Davis, who was about the house to run on errands and bring wood, was attacked; sick five weeks; recovered.

Clark, aged eighteen, a neighbor to Fox No. 1, was attacked about the first of March; was sick three weeks; recovered; was waiting upon the sick at Fox's.

Miss Hamilton, aged sixteen, neighbor to Fox No. 1, was waiting upon the sick of that family; was taken sick in March; down three weeks; took relapse; was sick three weeks more; recovered.

A widow woman and daughter, and another person, a man about thirty years of age, were attacked. All were engaged about the house of Fox No. 1, and all recovered.

In reference to the origin of this epidemic little can be said. The information obtained was meagre. The young man was confined to a close school-room, badly ventilated. From particular inquiries made it was ascertained that this form of fever had never been known to be in that neighborhood. Intermittent fever had not prevailed there for a great many years, nor had there been remittent fever. In fact the year before, up to the time when these cases occurred, had been unusually healthy. The disease extended over but a mile and a half of country, and was confined to seven families. Thirty-five persons altogether were attacked, of these seven died. The idea of its contagiousness did not obtain at all when the disease first made its appearance, and it was only after a considerable number were attacked that the truth became too apparent any longer to doubt it. In reference to the symptoms common to all attacked, they may be briefly stated as follows: In the access, headache, languor, soreness in the limbs, chills and rigors, subsequently skin hot and dry, circulation accelerated, and all more or less troubled with cough. In most of the cases there was muttering delirum, coma vigil. Several were with difficulty retained in bed. From my inquiries I found that three or four were troubled with nausea and vomiting at the beginning of the attack. Diarrhœa occurred in most of the cases. Several had involuntary discharges; several profuse bloody discharges, while pains in the bowels and tympanites were prominent symptoms. Profuse sweating occurred in the families of Fox and Davis, and a fact not unworthy of note is, that the fatal cases were those that sweated least. The duration of the disease varied from three days to eight weeks. There were three relapses. But very little information could be obtained as to the treatment.

*Oxford, O.*, June 20, 1855.

